# Molecular network mechanism of Shexiang Huayu Xingnao granules in treating intracerebral hemorrhage

**DOI:** 10.1002/ibra.12131

**Published:** 2023-09-10

**Authors:** Ke‐Qian Liu, Xue Bai, Ji‐Lin Chen, Guo‐Jiao Chen, Muhammad Ameen Jamal, Yu‐Qi He

**Affiliations:** ^1^ Key Laboratory of Basic Pharmacology of Ministry of Education and Joint International Research Laboratory of Ethnomedicine of Ministry of Education, School of Pharmacy Zunyi Medical University Zunyi China; ^2^ Department of Neurology South West Medical University Luzhou China; ^3^ Animal Center Kunming Medical University Kunming China; ^4^ Department of Theriogenology, Faculty of Veterinary Sciences University of Veterinary and Animal Sciences Lahore Pakistan

**Keywords:** behavioral function, intracerebral hemorrhage, network pharmacology, Shexiang Huayu Xingnao granules

## Abstract

We aim to explore the pharmacological efficacy and molecular network mechanism of Shexiang Huayu Xingnao granules (SX granules) in the treatment of intracerebral hemorrhage (ICH) based on experiments and network pharmacology. After the ICH model establishment, the behavioral functions of rats were assessed by the modified neurological severity score (mNSS), the wire suspension test, and the rotarod test. Brain histomorphological changes were observed using 2,3,5‐triphenyl tetrazolium chloride (TTC), hematoxylin–eosin (HE), Nissl, and TdT‐mediated dUTP nick end labeling (TUNEL) combined with neuronal nuclear (NEUN) immunofluorescence staining. The cross‐targets of SX granules and ICH were obtained using network pharmacology, gene ontology (GO) enrichment analysis, and Kyoto encyclopedia of genes and genomes (KEGG) signaling pathway analysis were performed. Then, the obtained Hub genes were verified using real‐time quantitative polymerase chain reaction (RT‐qPCR). The mNSS score was reduced and the duration to remain wire suspended increased in the SX group. In the morphological experiment, SX granules reduced brain tissue damage, neuronal apoptosis, and the number of astrocytes in the ICH rats. Moreover, 607 targets of drug–disease intersection were obtained by network pharmacology, and 10 Hub genes were found. SX granules regulated the expression of HRAS, MAPK3, and STAT3 in ICH condition. In conclusion, SX granules improved behavioral dysfunction, abnormal alterations in brain tissue, and cell morphology in ICH rats, and potential molecular mechanism was linked with the expression of multiple genes.

## Graphical abstract

This study investigated the efficacy and potential mechanism of Shexiang Huayu Xingnao granules (SX granules) in the treatment of intracerebral hemorrhage (ICH). Through behavioral tests, hematoxylin–eosin (HE), Nissl, immunofluorescence, and other research methods, we found that SX granules improved behavioral dysfunction and abnormal changes in brain tissue and cell morphology in ICH rats. We also used network pharmacology to explore the relevant targets and Hub genes of SX granules acting on ICH and further analyzed the key pathways of the drug to treat the disease through GO enrichment and KEGG pathway. Ultimately, the Hub genes were validated using real‐time quantitative polymerase chain reaction (RT‐qPCR), thus revealing the potential mechanism of action of SX granules in the treatment of ICH.

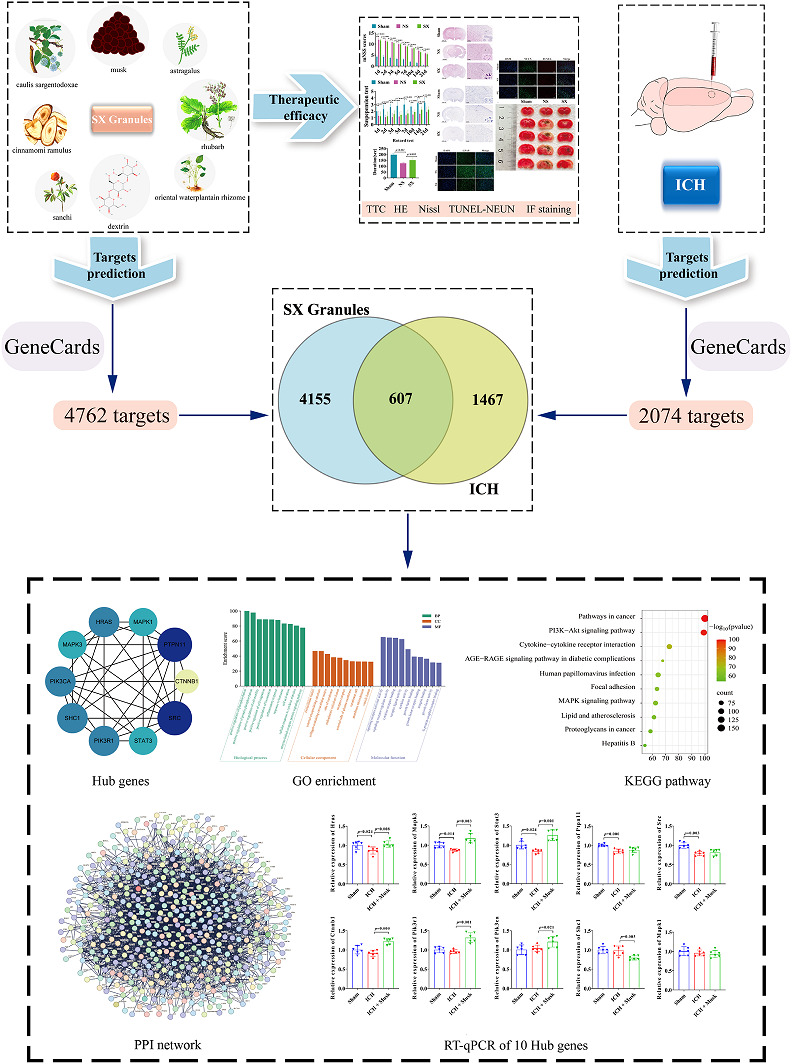

## 1 INTRODUCTION

Intracerebral hemorrhage (ICH) is a major killer that threatens human life and is associated with high morbidity, mortality, and poor prognosis.^1^, ^2^ According to etiology, risk factors, and anatomical site, ICH can be divided into primary and secondary hemorrhage, among which, ICH caused by injury of small cerebral vessels is primary hemorrhage, and ICH caused by specific related known etiology is secondary hemorrhage.^3^ About 85% are primary bleeding, but this is often associated with many diseases such as tumors, hypertension, and vascular malformations.^4^, ^5^ 30%–50% of patients with ICH have thrombus extension to the ventricles, obstructive hydrocephalus causes increased intracranial pressure, 5%–27% of patients develop delayed recurrent seizures or risk of epilepsy after ICH, 3%–7% of patients with ICH develop venous thromboembolism, approximately 60% of patients in the acute phase of ICH develop transient hyperglycemia, and over 70% develop hypertension.^6^, ^7^ For ICH treatment, early airway protection, hypertension control, anticoagulation therapy, and surgical intervention are the main means.^8^, ^9^ Meanwhile, stem cell therapy is also a promising approach for the treatment of ICH,^10^, ^11^ which corresponds to the usage of Chinese medicines, a challengeable hotspot from many reports.^12^

As a classic Chinese medicine for clinical treatment of ICH, Shexiang Huayu Xingnao granules (SX granules) are composed of musk, astragalus, *Cinnamomi ramulus*, rhubarb, sanchi, oriental water plantain rhizome, caulis sargentodoxae, and dextrin, which has been recognized with the effects of “wind medicine inducing resuscitation” and “activate blood and disinhibit water.”^13^ According to the previous study, SX granules significantly improve neurological function, reduce vasogenic and cytotoxic edema due to cerebral hemorrhage, and promote the repair of the blood–brain barrier on the side of the bleeding in rats with cerebral hemorrhage.^14^, ^15^, ^16^ In clinical application, SX granules have good efficacy, especially in the acute phase of ICH.^13^ However, its mechanism of action on ICH has not yet been clarified. In this study, we aimed to explore the mechanism of action of SX granules in the treatment of ICH and investigated the underlying molecular network mechanism by using network pharmacology method and biological experiment so as to provide some mechanism to understand the treatment of ICH with SX granules.

## 2 MATERIALS AND METHODS

### 2.1 Animal grouping

The animal study was legally approved by the Animal Care & Welfare Committee of Kunming Medical University with the approval number: KMMU2023MEC057. All animal surgeries were performed in accordance with the Declaration of Helsinki. SPF healthy male Sprague‐Dawley rats (weighing 180–220 g) were provided by the Experimental Animal Center of Kunming Medical University and randomly divided into three groups (*N* = 10/group): Sham group (Sham), ICH model treated with normal saline group (NS), and SX granules treatment group (SX). The SX group was given 0.16 g/100 g SX granules (The Affiliated Hospital of Traditional Chinese Medicine, Southwest Medical University, Batch number: 20220622) one time a day, last 3 weeks by gavage, and the other groups were given the same volume of normal saline.

### 2.2 ICH model

After fasting for 8 h at the age of 7 days, rats in NS and SX granules groups were anesthetized by intraperitoneal injection of pentobarbital sodium (40 mg/kg). Once skin preparation was sterilized, the rats were fixed in the prone position. A median longitudinal incision of approximately 2 cm was made, a point was taken 3 mm right of the midline of the anterior fontanel, and holes were drilled in the skull and dura along the sagittal plane. Then 50 μL of autologous arterial blood was slowly infused for about 2 min, and the needle was retained for 10 min to prevent blood backflow. Subsequently, bone wax was used to seal the bony foramen of the skull, and the scalp was sutured layer by layer. Finally, the wound was kept disinfected, and all rats were placed on a 37°C heating pad till the animal woke up. No surgical operations were performed in the Sham group.

### 2.3 Modified neurological severity score (mNSS)

To evaluate the recovery of neurological function after brain injury in rats, motor, sensory, reflex, and balance tests were performed. The mNSS scores were performed at 1, 2, 3, 5, 7, 10, 14, and 21 days after surgery, and the scoring criteria are shown in Table 1.

**Table 1:** mNSS scoring criteria.

Evaluation item	Behavior	Score
Tail lifting experiment	Normal forelimb and hindlimb	0
	The angle of the head moving greater than 10° basing on the vertical axis	1
	Forelimb flexion	1
	Hindlimb flexion	1
Motor function	Normally walking	0
	Cannot walk straight	1
	Constant circling toward the paretic side	2
	Tumbling toward the paretic side	3
Beam experiment	Staying and walking parallel to the beam	0
	Staying and scratching the beam	1
	Staying on the beam with one hindlimb hanging	2
	Staying on the beam with hindlimbs hanging	3
	Falling off with an attempt to stay on the beam for more than 40 s	4
	Falling off with an attempt to stay on the beam for more than 20 s	5
	Falling off with an attempt to stay on the beam within 20 s	6
Sensory test	Orienteering test (vision and tactile)	1
	Proprioception test (deep feeling)	1
Reflex and abnormal motion	Auricular reflex (head shaking when stimulating the ear canal)	1
	Corneal reflex (blinking when touching the cornea with a cotton swab)	1
	Startle reflex (moving when hearing short and sharp voice)	1
	Epilepsy or dystonia	1

Abbreviations: mNSS, modified neurological severity score; s, second.

### 2.4 Wire suspension test

The wire was fixed to the rod, a plastic basket was placed under the wire, and the rat was lifted to the wire 0.4 meters above the ground at 1, 2, 3, 5, 7, 10, 14, and 21 days after surgery. The rats were scored according to the time from the beginning to the landing and the behavioral state of the rat, and the specific scoring criteria were as follows: 0: unable to grasp the rope; 1: grab the rope with one or two claws; 2: can jump up (upward trend) and grab the rope with two claws; 3: can grasp the rope with 3 or 4 claws and roll up the tail; 4: crawl at least 30 cm on the rope; 5: successfully crawling from the starting point to the end of the rope and getting off the rope.

### 2.5 Rotarod test

The speed of the rotary rod was set at 30–35 rpm, and the rats in each group were trained adaptively for 3 days for 10 min per day. Then, the rotary rod speed was set to accelerate from 3 to 30 rpm in 3 min, and the time the rats spent on the rotary rod was recorded, repeated three times, and the longest time was recorded.

### 2.6 Sample harvest

The rats were anesthetized by inhaling isoflurane on the 21st day after surgery, and subsequently underwent an open thoracotomy. After exposure of the heart, 400 mL of normal saline was infused, followed by 500 mL of 4% paraformaldehyde fixative. Brains were rapidly removed and postfixed with 4% paraformaldehyde. Cortical tissue not infused and fixed with 4% paraformaldehyde was taken for real‐time quantitative polymerase chain reaction (RT‐qPCR).

### 2.7 2,3,5‐triphenyl tetrazolium chloride (TTC) staining

On the 21st day after surgery, rats were anesthetized with isoflurane (R580, RWD Lifescience Co., Ltd), the brains were taken directly, wiped clean of blood, placed in the center of the tray, and placed in the refrigerator at −80°C for 20 min, then removed, coronal sections (thickness about 2 mm) were made at −20°C, and TTC staining solution (409E033, Servicebio Co., Ltd) was added and placed in a 37°C thermostat for 20 min to avoid light staining. After the staining was finished, the TTC staining solution was carefully aspirated, and an appropriate amount of 4% paraformaldehyde was added and fixed overnight in the refrigerator at 4°C, then removed for photographing. The infarct ratio of each section was measured with ImageJ 1.4 software (National Institutes of Health, Bethesda, MD, USA). Infarct ratio = (contralateral hemisphere volume ‐ non‐ischemic ipsilateral hemisphere volume)/contralateral hemisphere volume.

### 2.8 Hematoxylin and eosin (HE) staining

The brain tissue of rats at 21 days after surgical operation was collected and immersed in 4% paraformaldehyde, then fixed for 72 h and performed paraffin embedding. Next, paraffin sections with a thickness of about 2 μm were prepared, deparaffinized, hydrated, and stained with HE staining solution (C0105, Beyotime Biotech Co., Ltd). Finally, the morphological changes in the cortex were observed under a microscope (CX40, Shunyu).

### 2.9 Nissl staining

After paraffin sections were deparaffinized, they were stained with 30 μL of 1% cresyl violet solution (G1430, Solarbio, Life Sciences Co., Ltd) in a humidified box in an incubator at 37°C for 9 min. After washing with distilled water, Nissl differentiation solution was added to the sections for 2 min, followed by the addition of 95% ethanol for rapid differentiation until Nissl bodies were purple and other tissues were colorless. Finally, they were dehydrated with absolute ethanol, transparent with xylene, and sealed with neutral glue. The images of the cortex were captured under a light microscope (Leica) at 20X magnification, with three fields of view in each section. Subsequently, the counts for both dark neurons and total neurons were conducted separately. Each sample underwent a repetition of 9 sections.

### 2.10 TdT‐mediated dUTP nick end labeling with neuronal nuclear (TUNEL‐NEUN) staining

After paraffin sections were deparaffinized, hydrated, and antigen repaired, 5% sheep serum (SL038, Solarbio, Life Sciences Co., Ltd) (mixed with 0.3% TritonX‐100, S‐T8787‐50ML, Sigma‐Aldrich) was added and blocked for 3 h at room temperature. Then, the primary antibody (NEUN, Rabbit, 1:200, GeneTex) was added and incubated for 16–18 h at 4°C. After washing, the slides were incubated with the secondary antibody (Goat Anti‐Rabbit Dylight 488, 1:400, Abbkine, California, USA) at room temperature for 3 hours. Following this incubation, the sections were thoroughly rinsed to remove any unbound secondary antibody. Subsequently, the TUNEL reaction was carried out by adding the TUNEL reaction solution (Rs‐11684817910, Roche, Shanghai, China) and incubating at room temperature for half an hour to label apoptotic cells. Finally, 4,6‐diamino‐2‐phenyl indole (DAPI) staining solution [DAPI (C1005, Beyotime Biotech Co., Ltd): anti‐quench agent (anti‐quench agent, P0126, Beyotime Biotech Co.) = 1:3000] was added to counterstained, and the morphological changes were observed and photographed under a fluorescence inverted microscope (Leica DMI6000B). Images were acquired using a 20X objective and the total number of TUNEL‐NEUN positive neurons was counted in three different regions of the cortex in each slice. Each sample was repeated in 9 slices.

### 2.11 Immunofluorescence staining

After paraffin sections were deparaffinized, hydrated and antigen repaired, 5% sheep serum (SL038, Solarbio, Life Sciences Co., Ltd) and 0.3% Triton X‐100 (S‐T8787‐50ML, Sigma‐Aldrich) were added for 3 h and incubated with primary antibody (Glial fibrillary acidic protein, GFAP, GB12090‐100, Mouse, 1:500, Servicebio) overnight at 4°C. Then, the sections were incubated with fluorescent dye‐labeled secondary antibody (Alexa Fluor 488 conjugated Goat Anti‐Mouse IgG, GB25301, 1:200, Servicebio) for 1 h at 37°C. After washing with PBST, the sections were blocked with a blocking agent (P0126, Beyotime Biotech Co., Ltd). Finally, the sections were observed under a fluorescence microscope. The density of GFAP‐immunoreactive astrocytes was measured in three different regions of the cortex for each section using ImageJ 1.4 software (National Institutes of Health) counting.

### 2.12 RT‐qPCR

The total RNA from the cortex at 28 days was extracted using Trizol reagent (Takara Bio Inc.) and reverse‐transcribed using RevertAid™ First Strand cDNA Synthesis System (Invitrogen). RT‐qPCR were carried out with Power SYBR (DBI Bioscience) according to the manufacturer's instructions. The expression level of each gene was normalized to that of GAPDH using the 2^−ΔΔ*ct*^ method. The primers used for the reaction are in Table 2.

**Table 2:** Primers sequence.

Gene	Sense primer	Antisense primer
GAPDH	GACATGCCGCCTGGAGAAAC	AGCCCAGGATGCCCTTTAGT
HRAS	GATCCCACTATAGAGGACTCC	TGCTGTGTCTAAGATGTCCA
MAPK1	CGCTTCAGACATGAGAACAT	CTGTGTCTTCAAGAGCTTGT
MAPK3	GAACCCCACCCCATTTTC	TCCACATCCAATCACCCA
PTPN11	CAGGTCCTAGGCACAGGAACTG	ACATTCCCAAATTGCTTGCCT
CTNNB1	TATGAGTGGGAGCAAGGC	CTGCGTGGATGGGATCT
SRC	CGAGGAAGGTGGATGTCAG	CCCGTCTAGTGATCTTGCC
STAT3	GCTGGAACAGCATCTTCAGG	CTGTCTGGTCACAGACTGGT
PIK3R1	GTCCTTAGCTCAGTACAATCC	TTGACAACTTGATCCTGCTG
SHC1	GAATGCCGATCACTCTCAC	TGATGGTTGGCAATGATCTG
PIK3CA	CTGCAGTTCAACAGCCACAC	ACAGGTCAATGGCTGCATCA

### 2.13 Drug targets acquisition

In the GeneCards database (https://www.genecards.org/), we entered the keywords of drug names in the prescription of SX granules: musk, astragalus, *Cinnamomi ramulus*, rhubarb, sanchi, oriental water plantain rhizome, caulis sargentodoxae, and dextrin, to obtain the target points of each drug component.

### 2.14 Disease targets acquisition

A keyword for the disease, “Intracerebral hemorrhage,” was input into the GeneCards database to obtain and download all disease targets.

### 2.15 Venn diagram

Drug and disease targets were entered in the Draw Venn Diagram platform (http://bioinformatics.psb.ugent.be/webtools/Venn/) to filter out drug–disease intersection targets so as to obtain a Venn intersection diagram of drugs and diseases.

### 2.16 Protein–protein interaction networks (PPI)

Under Multiple proteins on the String website (https://cn.string-db.org/), the drug–disease intersection targets were entered, then selected “Homo sapiens,” and clicked “SEARCH” for analysis so as to obtain the drug–disease PPI network interaction map.

### 2.17 Hub genes screening

Ten Hub genes were filtered by importing PPI network analysis from Cytoscape 3.8.0 software and using cytoHubba plugin with degree values.

### 2.18 Gene ontology (GO), Kyoto encyclopedia of genes and genomes (KEGG)

GO [biological processes (BP), cellular components (CC), and molecular functions (MF)] and KEGG analyses were performed in the Metascape database (https://metascape.org/gp/index.html#/main/step1) using drug–disease intersection targets, and the relevant compressed packages were downloaded.

### 2.19 Statistical analysis

Image beautification was performed using Cytoscape (Cytoscape Consortium, Version 3.8.0) and Adobe Illustrator software (Adobe Inc., Version 2020). All data were analyzed by one‐way ANOVA using SPSS software (IBM SPSS Statistics for Windows, Version 26.0). Data were presented as a mean ± s.e.m. (standard error of mean). The LSD test was used when the variance was homogeneous, and the Tamheri test was used when the variance was not homogeneous. *p* < 0.05 was considered statistically significant.

## 3 RESULTS

### 3.1 Behavioral evaluation of SX granules in improving ICH rats

**Figure 1:**
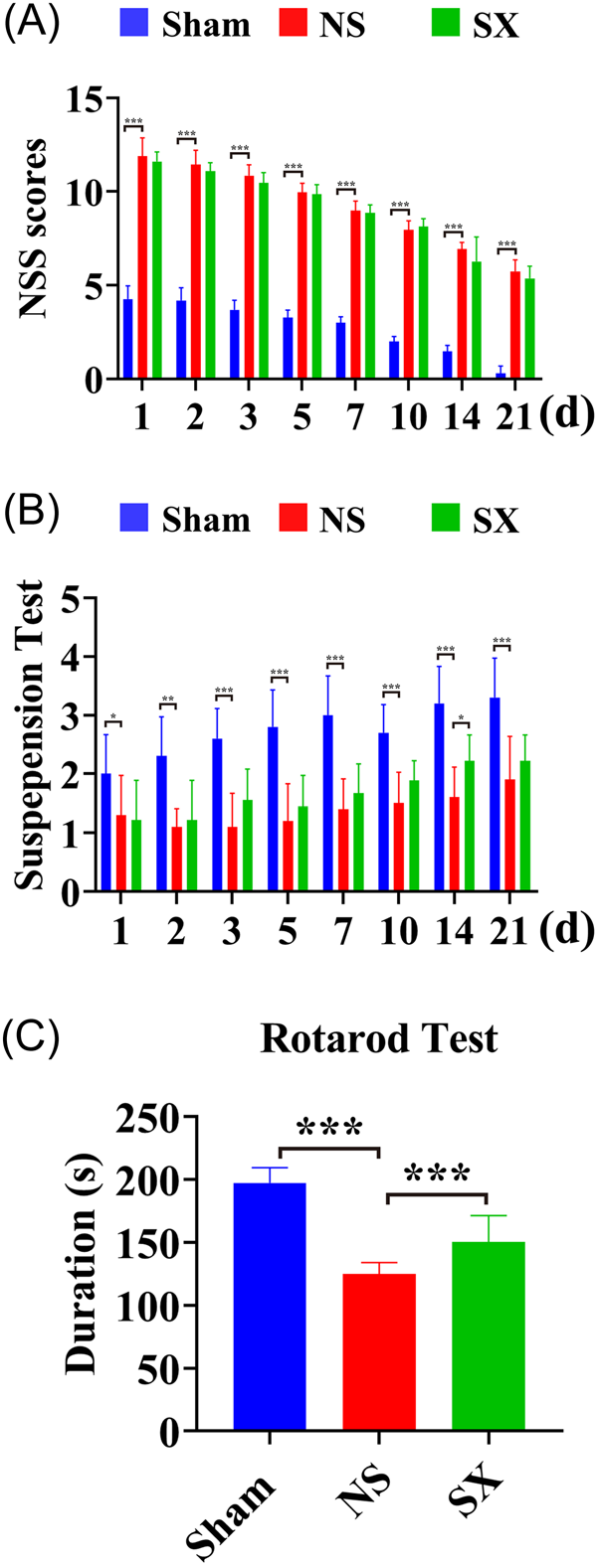
The results of behavioral experiments. (A) The mNSS scores for each group at 1, 2, 3, 5, 7, 10, 14, and 21 days postoperatively. (B) Wire suspension test scores for each group at 1, 2, 3, 5, 7, 10, 14, and 21 days postoperatively. (C) Duration on rotating rod of each group at 10 days postoperatively. d, day; mNSS, Modified Neurological Severity Score; NS, model group; s, second; Sham, sham group; SX, SX granules treatment group. *p < 0.05; **p < 0.01; ***p < 0.001. [Color figure can be viewed at wileyonlinelibrary.com]

The mNSS, wire suspension, and rotarod tests were used to evaluate the effect of ICH model on the behavioral function of rats. The mNSS scores showed that the scores of each group gradually decreased over time, and the scores of the NS group increased compared with the Sham group, the difference was statistically significant (Figure 1A, *p* < 0.001), while the scores in the SX group decreased compared with the NS group at Days 1, 2, 3, 5, 7, 14, and 21, but it exhibited no statistical significance (Figure 1A). Comparatively from the wire suspension test, the scores of the NS group were significantly lower than that of the Sham group (Figure 1B, *p* < 0.001), and the score of the SX group increased at Days 2, 3, 5, 7, 10, 14, and 21 and significantly increased at Day 14 when compared with the NS group (Figure 1B, *p* = 0.019). In the rotarod test, the NS group had a significantly shorter duration than the Sham group (Figure 1C, *p* < 0.001), while the SX group had a significantly longer duration than the NS group (Figure 1C, *p* = 0.001).

### 3.2 SX granules treatment ameliorates brain damage in ICH rats

**Figure 2:**
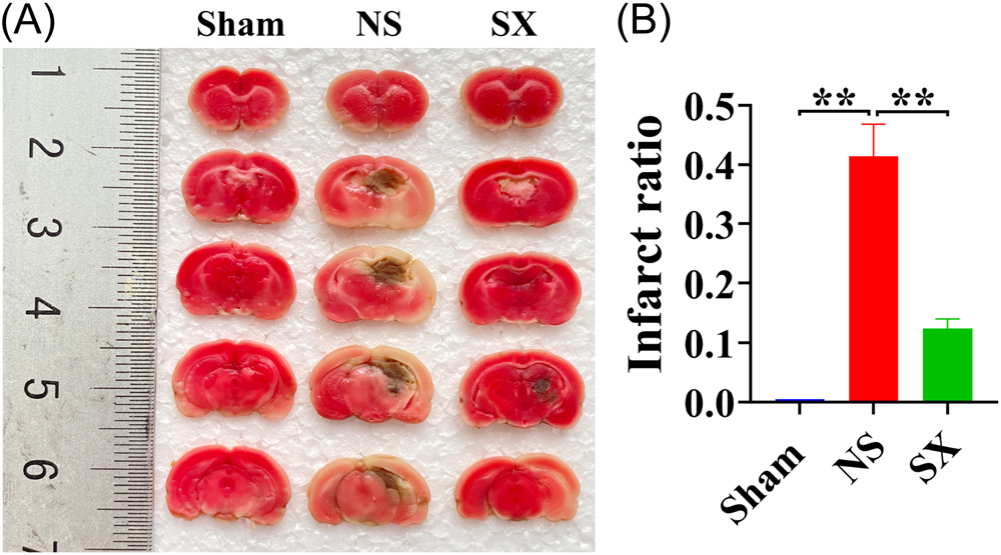
The results of TTC staining. (A) The results of TTC staining. (B) The results of infarct ratio in TTC staining. NS, model group; Sham, sham group; SX, SX granules treatment group; TTC, 2,3,5‐triphenyl tetrazolium chloride. **p < 0.01. [Color figure can be viewed at wileyonlinelibrary.com]

According to the staining results, ICH could result in severe brain tissue damage. Compared with the Sham group, the NS group had a reduction in the red area of brain tissue and a significant increase in the infarct area (*p* = 0.005). Furthermore, the red area increased, and the infarct area was significantly reduced in the SX group when compared with the NS group (Figure 2A,B, *p* = 0.020).

### 3.3 SX granules treatment improves cerebral pathological changes in ICH rats

**Figure 3:**
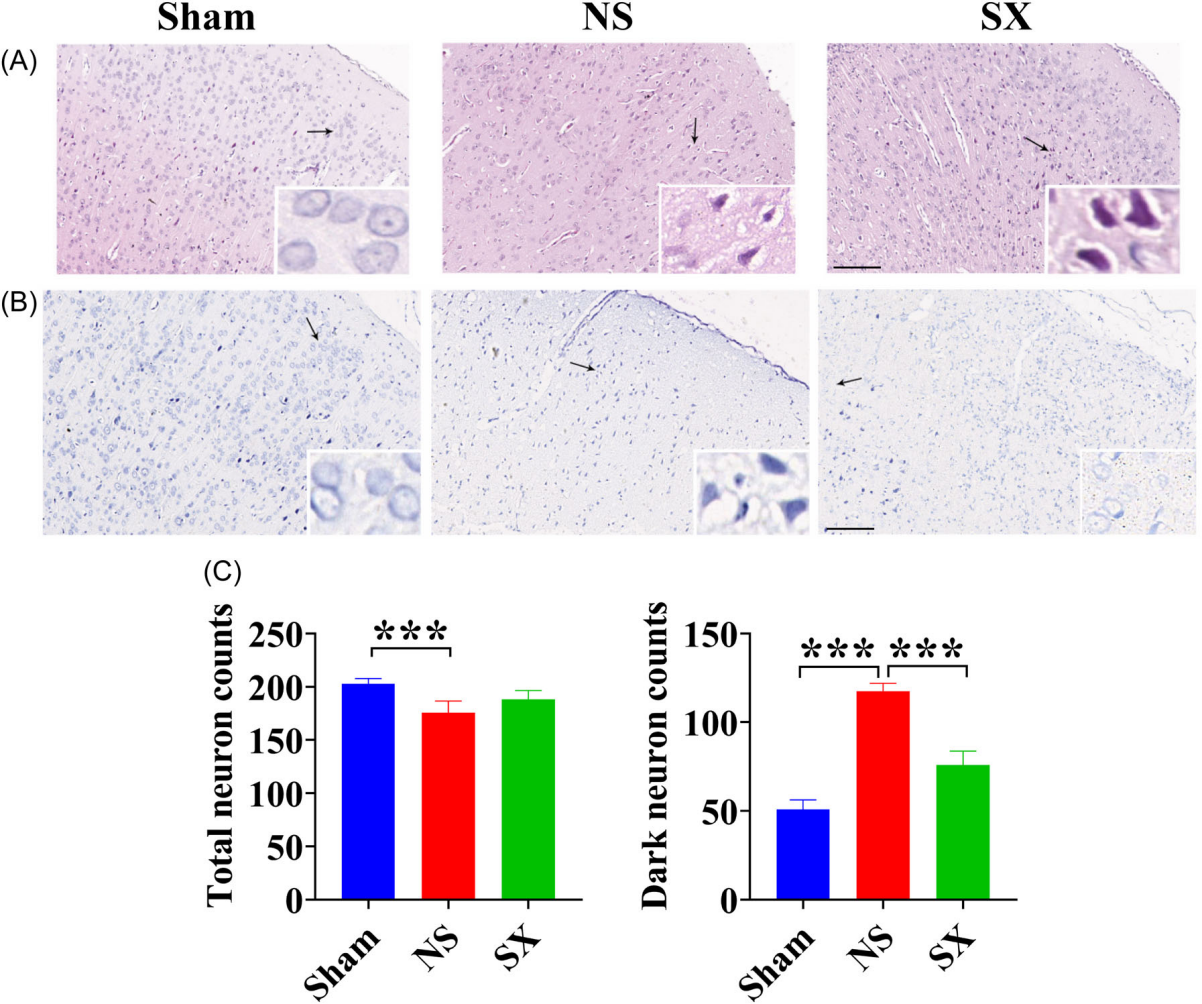
The results of HE staining and Nissl staining in rat cortex. (A) HE staining results for each group. Scale bars, 50 μm. Black arrows indicate neuronal cells. (B), (C) Nissl staining and quantitative histograms of neuronal cell counts results for each group. Scale bars, 50 μm. Black arrows indicate neuronal cells. HE, Hematoxylin and Eosin; NS, model group; Sham, sham group; SX, SX granules treatment group. ***p < 0.001. [Color figure can be viewed at wileyonlinelibrary.com]

By observing HE staining and Nissl staining, the results showed that the morphology of tissues and cells in the Sham group was normal and the structure was clear, while the NS group showed obvious tissue loss, abnormal cell morphology, and inflammatory factor infiltration, which is corresponding to the number of neuronal cells decreased and the number of apoptotic cells increased (Figure 3A). However, tissue and cell morphology were restored, inflammatory factor infiltration was reduced, the number of neuronal cells was increased, and the number of apoptotic cells was decreased in the SX group, compared with the NS group (Figure 3B,C).

### 3.4 SX granules treatment improves neuronal apoptosis in ICH rats

**Figure 4:**
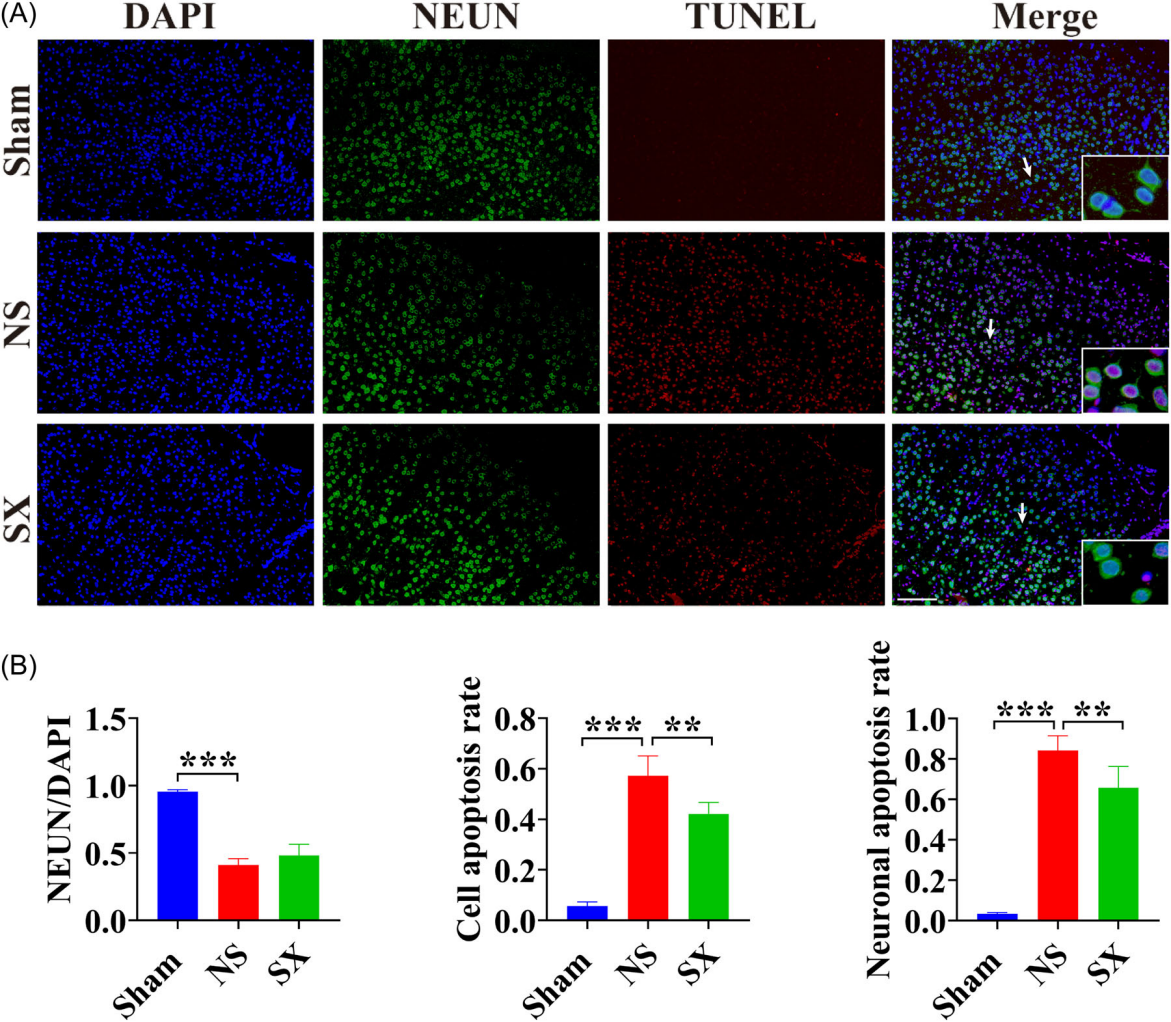
The results of TUNEL‐NEUN staining in rat cortex. (A) TUNEL‐NEUN staining results for each group. Scale bars, 50 μm. White arrows indicate neuronal cells. (B) The results of NEUN/DAPI, cell apoptosis rate and neuronal apoptosis rate for each group. DAPI, 4,6‐diamino‐2‐phenyl indole; NS, model group; Sham, sham group; SX, SX granules treatment group; TUNEL‐NEUN, TdT‐mediated dUTP Nick End Labeling with Neuronal Nuclear. **p < 0.01; ***p < 0.001. [Color figure can be viewed at wileyonlinelibrary.com]

As a neuronal cell marker, NEUN can be used to recognize neuronal cells, and apoptosis could be observed using TUNEL staining. As a result, it can be seen that the number of neurons decreased and the number of apoptotic cells increased in the NS group, compared with the Sham group (Figure 4A,B, *p* < 0.001). Moreover, the number of neuronal cells increased and the number of apoptotic cells decreased in the SX group, compared with the NS group (Figure 4A,B, *p* < 0.01).

### 3.5 SX granules therapy alleviates GFAP‐mediated inflammatory response

**Figure 5:**
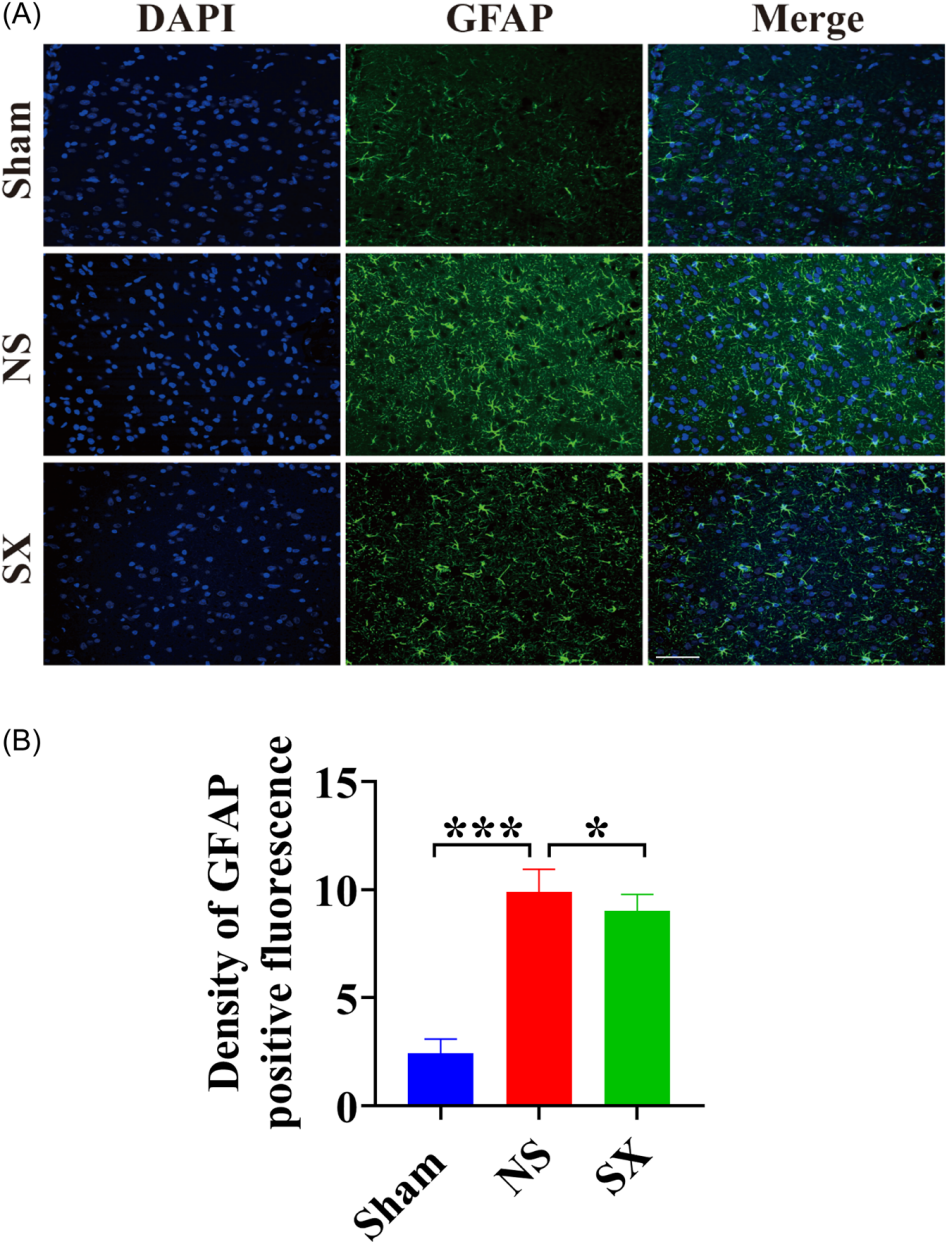
The results of immunofluorescence staining. (A) Immunofluorescence staining results of GFAP for each group. Scale bars, 50 μm. (B) The results of density of GFAP positive fluorescence for each group. DAPI, 4,6‐diamino‐2‐phenyl indole; GFAP, Glial fibrillary acidic protein; NS, model group; Sham, sham group; SX, SX granules treatment group. *p < 0.05; ***p < 0.001. [Color figure can be viewed at wileyonlinelibrary.com]

According to the staining results, when compared with the Sham group, the number of astrocytes and the fluorescence intensity were all enhanced in the NS group (Figure 5A,B, *p* < 0.001). However, there were fewer astrocytes and lower fluorescence intensity in the SX group than in the NS group (Figure 5A,B, *p* = 0.036).

### 3.6 Screening on active components of SX granules and targets of ICH disease

**Figure 6:**
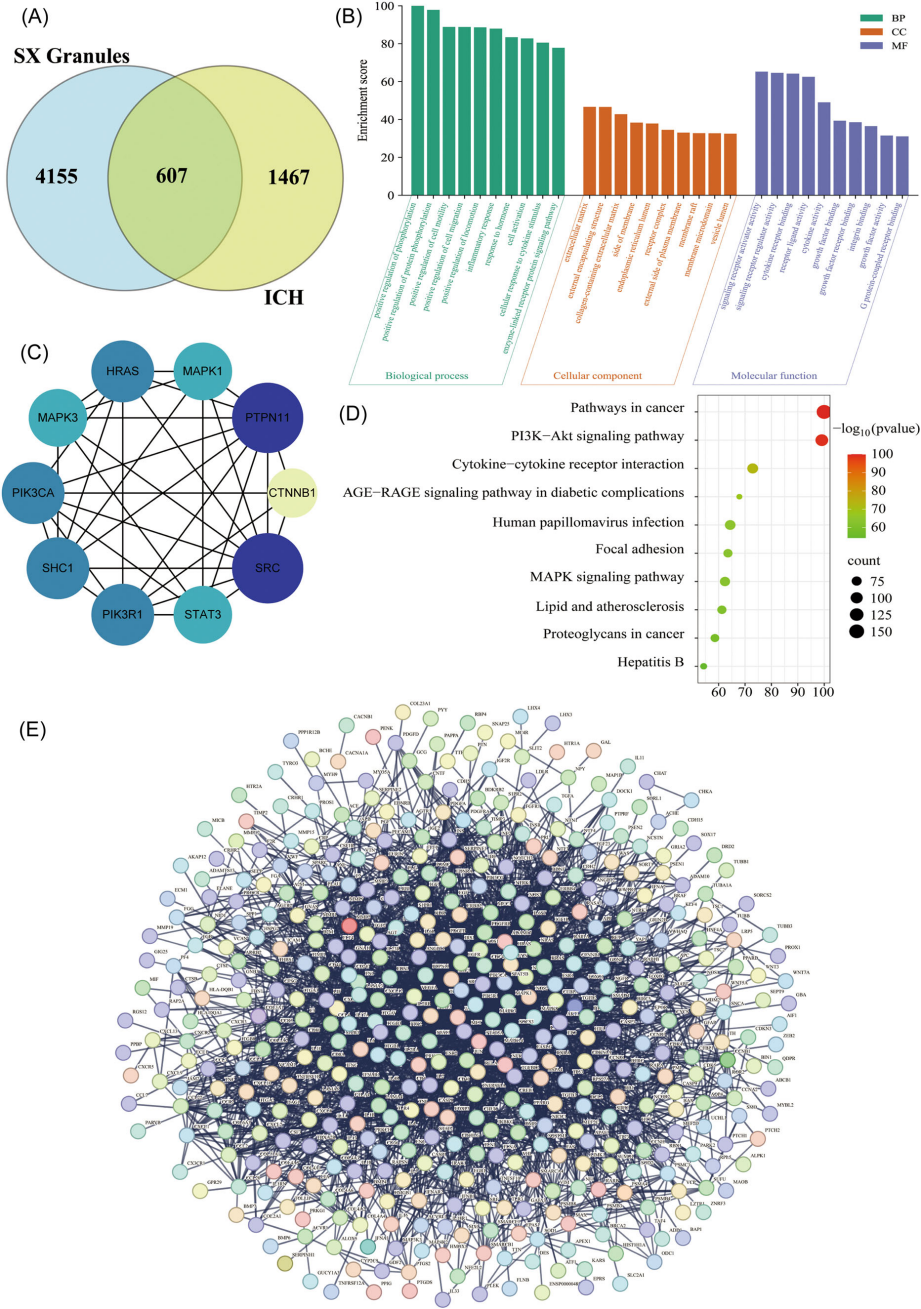
Construction of a network relationship map between SX granules and ICH using network pharmacology approaches. (A) The intersection targets of drug and disease. (B) Results of GO enrichment analysis. (C) Hub gene screening results. The larger the degree value, the larger the circle and the darker the color. (D) Results of KEGG signaling pathway analysis. (E) The PPI network interactions map. BP, biological process; CC, cellular component; ICH, intracerebral hemorrhage; MF, molecular function. [Color figure can be viewed at wileyonlinelibrary.com]

Four thousands seven hundred and sixty‐two drug targets (de‐duplicated) and 2074 disease targets were screened using GeneCards database (Figure 6A). Among the drug targets, the component targets were 4625 for musk, 142 for astragalus, 82 for rhubarb, 6 for *Cinnamomi ramulus*, 5 for sanchi, 2 for caulis sargentodoxae, 1 for oriental water plantain rhizome, and 69 for dextrin. In addition, 607 intersection targets were obtained using the Draw Venn Diagram platform (Figure 6A).

#### 3.6.1 PPI network construction and screening of core targets

PPI network interactions were constructed by PPI network relationships (Figure 6E), and the 10 most core targets (Hub genes) of SX granules and ICH were obtained from the analysis results of Cytoscape software, which were SRC, PTPN11, HRAS, PIK3R1, PIK3CA, SHC1, STAT3, MAPK1, MAPK3, and CTNNB1 in descending order according to the degree value (Figure 6C).

#### 3.6.2 GO function enrichment and KEGG pathway analysis of core target genes

GO enrichment analysis yielded 214 BP, 83 CC, and 72 MF. BP was mainly enriched in the positive regulation of phosphorylation processes, CC was mainly enriched in the extracellular matrix, and MF was mainly involved in signaling receptor activator activity (Figure 6B). KEGG enriched 115 relevant pathways, with bubble plots to show 10 of the more highly correlated signaling pathways (Figure 6D). Among the pathways associated with proliferation, differentiation, and apoptosis are signaling pathways such as MAPK, PI3K‐Akt, and Focal adhesion.

### 3.7 Changes in mRNA expression levels in the posterior cortex of ICH rats treated with SX granules

**Figure 7:**
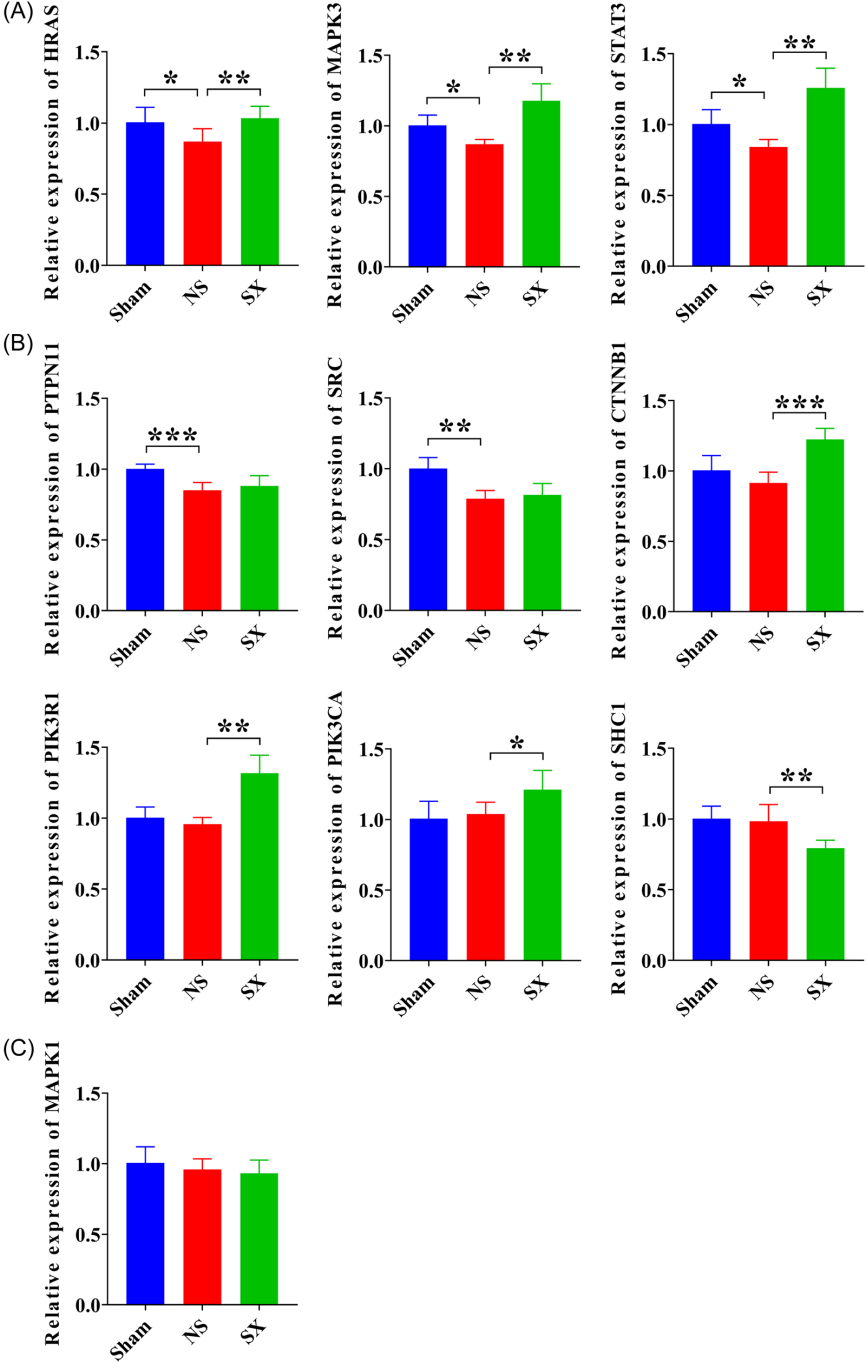
Relative mRNA expression changes of 10 Hub genes detected by RT‐qPCR. (A) Genes with decreased expression after brain hemorrhage injury and increased expression after SX Granules treatment. (B) Genes with changes after injury or differences after treatment. (C) Genes with no difference. NS, model group; Sham, sham group; SX, SX granules treatment group. *p < 0.05; **p < 0.01; ***p < 0.001. [Color figure can be viewed at wileyonlinelibrary.com]

The RT‐qPCR results showed that treatment of cerebral hemorrhage after SX granules significantly restored the expression of HRAS (*p* = 0.008), MAPK3 (*p* = 0.003), STAT3 (*p* = 0.001), CTNNB1 (*p* < 0.001), PIK3R1 (*p* = 0.001), and PIK3CA (*p* = 0.021) genes in the NS group (Figure 7A,B). Whereas, in the NS group, PTPN11 (*p* = 0.000) and SRC (*p* = 0.003) genes showed a significant decrease, which was restored by the administration of SX granules (Figure 7B). Additionally, SHC1 showed a significant decrease in its mRNA expression compared to the NS group after administration of SX granules, while the MAPK1 gene did not show significant changes after ICH or treatment of SX granules (Figure 7B,C).

## 4 DISCUSSION

Modern medical research points out that ICH includes primary and secondary injuries. Primary injury refers to the mechanical compression of the surrounding tissues caused by hematoma formation, which results in ischemia and hypoxia in the local tissues of the hemorrhage, which can form a placeholder effect and triggering structural damage.^17^ Secondary injury is mainly a series of rapid events induced in the cells affected by this initial injury, such as excitotoxicity, oxidative stress, and mitochondrial disorders, all of which contribute to secondary brain injury.^18^ According to the available studies, the size of the hematoma formed by primary injury is a major determinant of outcome after cerebral hemorrhage, and the greater the volume of the hematoma, the greater the neurological damage, and the worse the prognosis.^19^, ^20^ Although the body's self‐protective response can limit the extent of brain damage and promote recovery, this response is often not very enough during the acute phase of hemorrhagic stroke. Therefore, accelerating hematoma absorption, ameliorating neuronal damage, and repairing the blood–brain barrier are the keys to treating cerebral hemorrhage disease. SX granules, a traditional Chinese medicine compound preparation, can reduce cytotoxic edema and vasogenic edema after cerebral hemorrhage and promote the repair of the blood–brain barrier on the hemorrhagic side, which has been used in the clinical treatment of ICH.^13^ Modern pharmacological studies have shown that SX granules have the ability to increase superoxide dismutase activity, decrease malondialdehyde level, inhibit matrix metalloproteinase‐9 expression, and increase brain‐derived neurotrophic factor (BDNF) expression level to protect the brain nerves, but the mechanism of action is not clear.^13^ Therefore, the aim of this study was to explore the mechanism of the therapeutic action of SX granules for the treatment of ICH through pharmacological experiments and network pharmacology.

PPI network analysis of potential genes for ICH treatment with SX granules yielded 10 core targets, suggesting that these genes may be key targets for the treatment of ICH, and almost all of the key targets are related to cell proliferation, differentiation, and survival. Further, GO enrichment analysis of the core targets showed that SX granules for the treatment of ICH mainly act on the positive regulation of biological processes of protein phosphorylation, the molecular function of signaling receptor activators, and the cellular components of the extracellular matrix. KEGG signaling pathway analysis revealed that the highest proportion of them was the pathways in cancer. The two most important pathways in this pathway are PI3K/AKT/mTOR and Ras/MAPK, which are tightly interconnected through a number of positive and negative feedback loops.^21^ These results suggest that SX granules most likely act to treat ICH through several biological processes, targets, and pathways, and that the specific mechanisms need to be further elucidated.

HRAS is one of the three major proto‐oncogenes of the RAS gene family, and MAPK3, as a member of the MAP kinase family, has been known to be involved in the regulation of Ras and ERK proteins, respectively. Also, MAPK can regulate a wide range of important aspects of cellular growth, differentiation, stress, and inflammatory response.^22^ The STAT3 gene is a key transcription factor in the JAK2/STAT3 signaling pathway, and activated JAK2 protein can induce tyrosine residues of downstream target proteins to enrich and phosphorylate STAT3 so that it enters the nucleus and binds to the target gene, thereby regulating cellular transcription. Activated JAK2 protein can induce tyrosine residues of downstream target proteins to enrich and phosphorylate STAT3 so that it enters the nucleus and binds to target genes, further regulating the transcription of downstream genes and thus regulating cell proliferation, differentiation, apoptosis, and immune response.^23^ It has been shown that inhibition of the MAPK pathway activates the JAK2/Stat3 pathway and that both pathways are regulated by receptor tyrosine kinase (RTK) and nonreceptor tyrosine kinase (NRTK).^24^ CTNNB1 belongs to the cysteine‐rich glycoprotein family (wingless, Wnt) signaling pathway, which is one of the important downstream effectors, and plays a key role in cell proliferation, apoptosis, and adhesion.^19^ In addition, HRAS, PIK3CA, and PIk3R1 genes are important coding genes for PI3K/AKT, a signaling pathway that is closely related to all stages of cell growth and development. Activation of HRAS, PIK3CA, and PIK3R1 genes has been reported to protect neuronal cells, vasodilate blood vessels, and ameliorate microcirculatory disorders.^25^, ^26^ Thus, the signaling pathways mediated by MAPK, Wnt, and PI3K/AKT are important in ICH. Signal transduction pathways mediated by MAPK, Wnt, and PI3K/AKT play an important role in the pathogenesis of ICH and regulate various oxidative stress and inflammatory processes in vivo.

The results of animal experiments showed that the behavior of ICH rats improved after intervention with SX granules, and the mRNA levels of HRAS, MAPK3, STAT3, CTNNB1, PIK3R1, and PIK3CA were significantly increased in the administered group compared with the model group. In histomorphology, HE staining, Nissl staining, TUNEL‐NEUN staining, and immunofluorescence staining of rats in the normal group did not show any abnormality; the tissues of rats in the model group showed obvious tissue loss, abnormal cellular morphology, and infiltration of inflammatory factors, as well as a decrease in the number of neuronal cells and an increase in the number of apoptotic cells and astrocytes. The intervention of SX granules showed that there was a restoration of the morphology of tissue cells and a decrease in the infiltration of inflammatory factors. After the intervention of SX granules, the tissue cell morphology was restored, the inflammatory factor infiltration was reduced, the number of apoptotic cells and astrocytes was reduced, and the number of neuronal cells was increased, which suggests that SX granules may be able to protect the brain tissue of ICH rats by activating the abovementioned cell proliferation pathway, inhibiting the inflammatory factor production pathway and apoptosis pathway to improve the oxidative stress injury in the brain and alleviate the disease state of the rats.

## 5 CONCLUSION

In summary, the treatment of ICH by SX granules has the characteristics of multicomponent, multitarget, and multipathway, which may regulate inflammatory signals and cell proliferation and differentiation‐related pathways such as MAPK, PI3K/AKT, Wnt, and JAK2/STAT3 through cell proliferation and differentiation and inflammation targets such as HRAS, MAPK3, CTNNB1, and STAT3, slowing down the ICH‐induced neuronal apoptosis and inflammatory response, thus exerting a protective effect on neurons. Together, this study may provide a certain reference basis for the research and clinical application of SX granules in the prevention and treatment of cerebral hemorrhage and its related symptoms. However, the theoretical results need to be verified by subsequent experiments.

## AUTHOR CONTRIBUTIONS

Xue Bai, Ji‐Lin Chen, and Guo‐Jiao Chen interpreted the data and arranged the figures. Ke‐Qian Liu wrote and revised the manuscript. Muhammad Ameen Jamal participated in the design and review of this study. Yu‐Qi He was involved in the overall design and supervision of the work. All authors have read and approved the final version of the manuscript.

## CONFLICT OF INTEREST STATEMENT

The authors declare no conflict of interest.

## ETHICS STATEMENT

The animal study was legally approved by the Animal Care & Welfare Committee of Kunming Medical University with the approval number: KMMU2023MEC057.

## Data Availability

The data used and/or analyzed during the current study are available from the corresponding author upon reasonable request.
